# New vision-incorporated third-generation video laryngeal mask airways for intubation of patients in prone position

**DOI:** 10.1007/s10877-022-00925-y

**Published:** 2023-03-16

**Authors:** Tomasz Gaszynski

**Affiliations:** Department of Anesthesiology and Intensive Therapy, Medical University of Lodz, Lodz, Poland

Editor,

Sudden unexpected extubation of a patient in the prone position is one of the critical events during general anesthesia. Proposals for an algorithm of airway management in accidental extubation in patients positioned in prone positions were proposed recently, underpinning the role of a supraglottic airway device (SAD) as a first-line approach. [1, 2] (Bosch / Gaszynski). Bosch et al. [1] proposed using the SAD as an intubation conduit but did not specify how it could be performed. Gaszynski’s algorithm added the use of a videolaryngoscope (VL) and of the option of fiberoptic intubation through SADs in the prone position. [2, 3] However, the use of a channeled hyperangulated blade VL in patient in the prone position requires operator experience. Fiberoptic intubation (not using supraglottic device in conjunct) in case of a critical situation of an unexpected extubation in a patient in the prone position is even more challenging than a videolaryngoscope attempt. Therefore, the option of fiberoptic intubation (if necessary) through an SAD in a patient in the prone position seems to be an easier alternative. Second generation SADs are constructed to allow for intubation through its lumen [4]. Recently, new vision-incorporated third-generation video laryngeal mask airways became available, which allow for intubation under vision [5, 6]. One such example is the SaCo Video Laryngeal Mask (VLM™, UE Medical®, Zhejiang, China) Fig. 1. Blind intubation through SADs in the prone position is not an acceptable technique due to the high failure rate [7] and therefore should be abandoned in favor of a direct-vision technique [5]. If SADs are used in suitable elective surgical cases with the patient in the prone position, it provides the option to intubate patient, if necessary, in the prone position without the need to change the patient position. In addition, the new new vision-incorporated third-generation video laryngeal mask airways allows for continuing oxygenation of to oxygenate the patient during the intubation efforts.

**Fig. 1:**
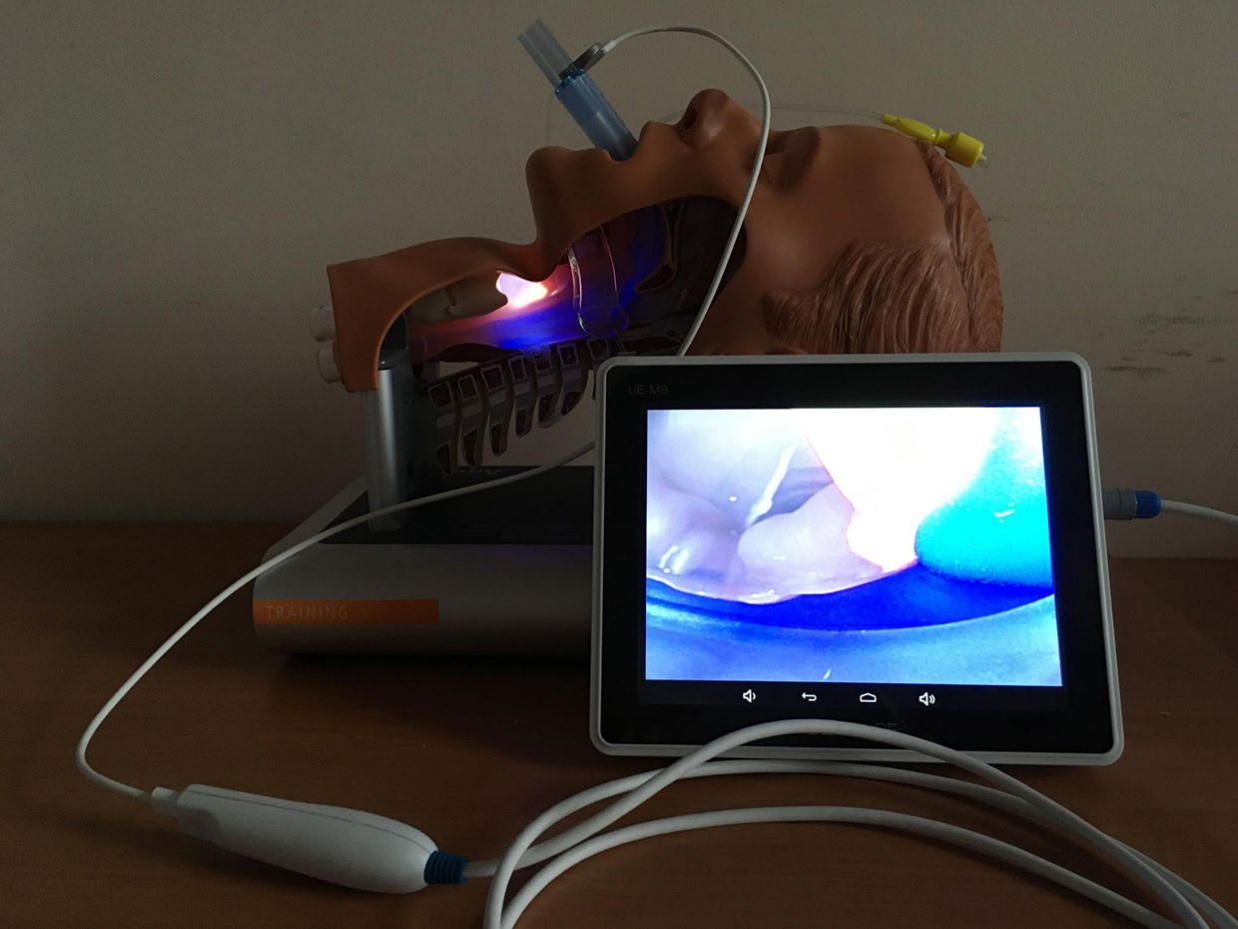
SaCo Video Laryngeal Mask (VLMTM, UE MedicalR, Zhejiang, China)

In this letter, a personal experience is described, in which an unexpected extubation occurred during elective spinal surgery with the patient in the prone position.

**Fig. 2:**
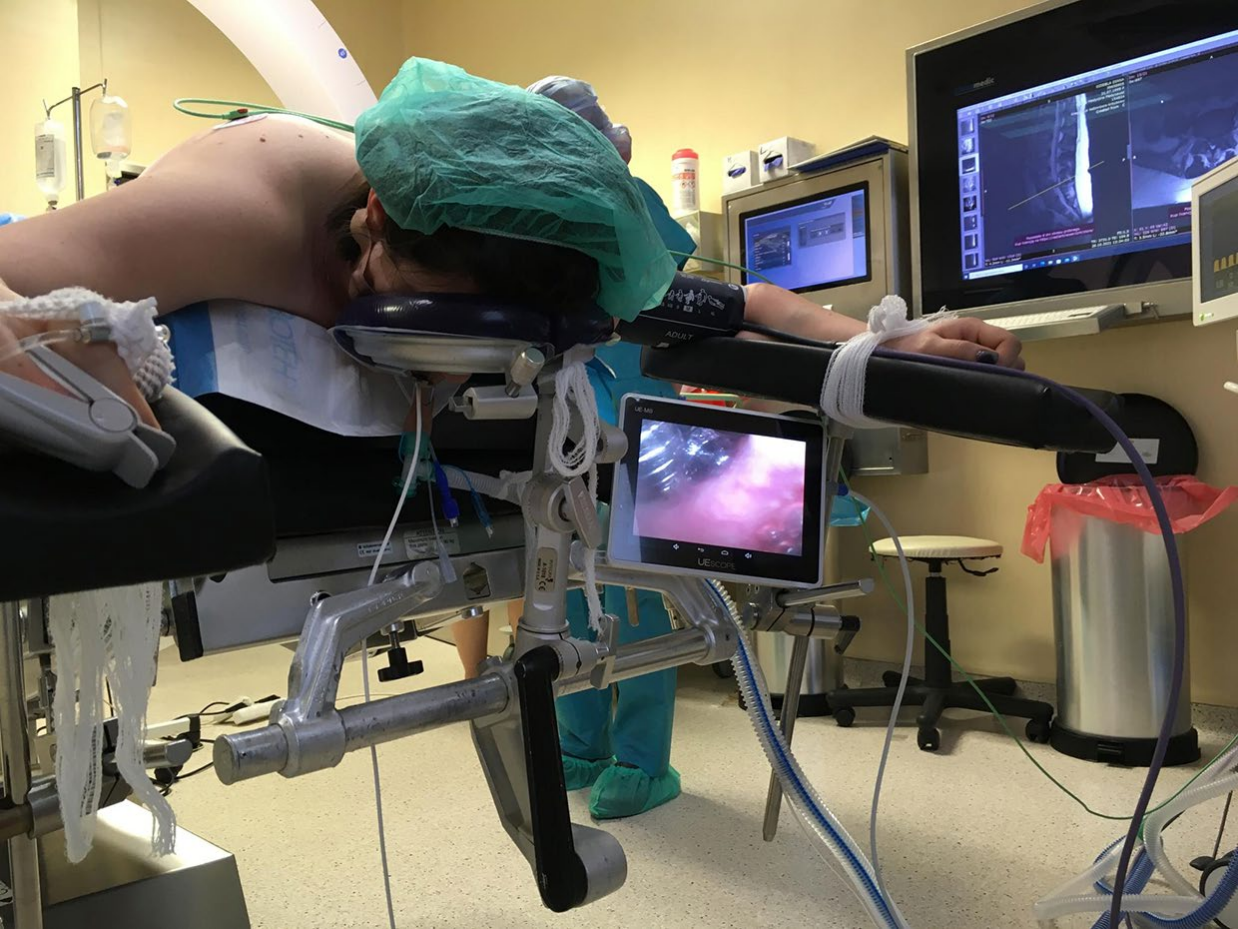
Use of SaCo Video Laryngeal Mask (VLMTM, UE MedicalR, Zhejiang, China) for intubation in patient positioned in prone position

Patient was anesthetized and intubated in supine position. Just after changing the position from supine to prone, before surgery started, a significant leak in the respiratory circuit was noticed, with a malfunctioning sealing of the SAD cuff. Because oxygenation was possible it was decided not to turn the patient to supine and intubate but remove the endotracheal tube in the prone position and introduce the VLM. After successful VLM placement and providing adequate ventilation, the patient was successfully intubated in the prone position using the VLM as intubation conduit (Fig. 2.). The procedure was successful on the first attempt without any complications. As an extra advantage of this technique, ventilation of the lungs is uninterrupted if prolonged intubation attempts would be necessary. There were no complications of airway procedure. Patient consent for publication of picture was obtained.

## In conclusion

This letter supports the alternative use of an SAD in case of an unexpected extubation in a patient positioned in the prone position as a first-line approach. The algorithms of management of a critical situation as a sudden extubation of a patient in the prone position may include an attempt of fiberoptic intubation through the SAD. The new vision-incorporated third-generation video laryngeal mask airways can be used for such purpose as an alternative to fiberoptic intubation.

## References

[1] Bosch L, Pacreu S, Castelltort L, Gallart L (2021). Accidental extubation in prone position. Report of two cases and proposal of an algorithm for airway management. Eur J Anaesthesiol.

[2] Gaszynski T (2020). Algorithm for management of sudden unexpected extubation in patient positioned in prone position. Anaesthesiol Intensive Ther.

[3] Gaszynski T (2019). Intubation in prone position using AirTraq Avant videolaryngoscope. J Clin Monit Comput.

[4] Cook TM. Third generation supraglottic airway devices: an undefined concept and misused term. Time for an updated classification of supraglottic airway devices. British Journal of Anaesthesia 2015;115(4):633–642. 10.1093/bja/aev309 26385672

[5] Van Zundert AAJ, Kumar CM, Van Zundert TCRV, Gatt SP, Pandit JJ (2021). The case for a 3rd generation supraglottic airway device facilitating direct vision placement. J Clin Monit Comput.

[6] Van Zundert AAJ, Gatt SP. Van Zundert TCRV et al. Features of new vision-incorporated third-generation video laryngeal mask airways. J Clin Monit Comput (2021). ahead of print 10.1007/s10877-021-00780-3. 10.1007/s10877-021-00780-3 34919170

[7] van Dijck M, Houweling BM, Koning MV (2020). Blind intubation through an i-gel® in the prone position: A prospective cohort study. Anaesth Intensive Care.

